# Strong Activity and No Resistance Induction Exerted by Cell-Free Supernatants from *Lacticaseibacillus rhamnosus* against Mono-Species and Dual-Species Biofilms of Wound Pathogens in In Vivo-like Conditions

**DOI:** 10.3390/ijms25042087

**Published:** 2024-02-08

**Authors:** Esingül Kaya, Marta Bianchi, Giuseppantonio Maisetta, Semih Esin, Giovanna Batoni

**Affiliations:** Department of Translational Research and New Technologies in Medicine and Surgery, University of Pisa, Via S. Zeno 37, 56123 Pisa, Italy; e.kaya@studenti.unipi.it (E.K.); marta.bianchi@phd.unipi.it (M.B.); giuseppantonio.maisetta@unipi.it (G.M.); semih.esin@unipi.it (S.E.)

**Keywords:** wound infections, *Pseudomonas aeruginosa*, *Staphylococcus aureus*, *Lacticaseibacillus rhamnosus*, resistance induction, wound model, postbiotics, cell-free supernatants, biofilm

## Abstract

It is widely agreed that microbial biofilms play a major role in promoting infection and delaying healing of chronic wounds. In the era of microbial resistance, probiotic strains or their metabolic products are emerging as an innovative approach for the treatment of hard-to-heal (chronic) wounds due to their antimicrobial, healing, and host immune-modulatory effects. In this study, we aimed to investigate the potential of cell-free supernatants (CFS) from *Lacticaseibacillus rhamnosus* GG against mono- and dual-species biofilms of wound pathogens in a 3D in vitro infection model. Mature biofilms of *Pseudomonas aeruginosa* and *Staphylococcus aureus* were obtained on collagen scaffolds in the presence of a simulant wound fluid (SWF) and treated with CFS at different doses and time intervals. At 1:4 dilution in SWF, CFS caused a marked reduction in the colony forming-unit (CFU) numbers of bacteria embedded in mono-species biofilms as well as bacteria released by the biofilms in the supernatant. CFU count and electron microscopy imaging also demonstrated a marked antibiofilm effect against dual-species biofilms starting from 8 h of incubation. Furthermore, CFS exhibited acceptable levels of cytotoxicity at 24 h of incubation against HaCaT cells and, differently from ciprofloxacin, failed to induce resistance after 15 passages at sub-inhibitory concentrations. Overall, the results obtained point to *L. rhamnosus* GG postbiotics as a promising strategy for the treatment of wound biofilms.

## 1. Introduction

Chronic non-healing wounds are a prevalent medical problem affecting millions of patients across the world [1]. These wounds often exist as comorbidities and include diabetic foot ulcers, venous leg ulcers, and pressure ulcers, thus being particularly predominant in elderly people [2]. With the general aging of the population, the burden of chronic wounds is destined to increase, having a major impact on healthcare resources in addition to the quality of life of many patients [3]. Chronic wounds are at a high risk of infection, with *Pseudomonas aeruginosa* and *Staphylococcus aureus* being among the most represented wound pathogens [4]. In most chronic wounds, infectious agents are found in the form of biofilms, which are complex and often multispecies bacterial communities embedded in a self-produced extracellular matrix [5]. It is widely agreed that the presence of biofilms plays a major role in the management of chronic wounds as it hampers the clearance by host immune response, the success of antimicrobial therapy, and the process of healing [5].

Besides aggressive debridement and irrigation with disinfectants, topical or systemic antibiotic therapy is part of the management of an infected wound [5,6]. Nevertheless, the unprecedented rise of bacterial antibiotic resistance markedly reduces the number of therapeutic options available, stimulating the search for nontraditional antimicrobial alternatives for the treatment of infected wounds [7,8,9].

Among these alternatives, the use of probiotics or their products is an emerging trend in wound care [10,11]. Such a trend is in agreement with what was stated by the Organization for Economic Cooperation and Development that suggested the use of probiotics as alternative treatments to antibiotics to avoid the emergence of resistant microorganisms [12]. Probiotics may antagonize pathogens by several mechanisms including interference with pathogen adhesion (via exclusion, competition, or displacement), inhibition of pathogen growth and virulence, enhancement of mucosal barrier integrity, and modulation of inflammation and immune response [13].

Of note, at least part of the antagonistic activity of probiotics against pathogens is due to their ability to produce and release a plethora of bioactive substances (e.g., organic acids, hydrogen peroxide, bacteriocins, and antimicrobial peptides) endowed with potent and rapid antimicrobial activity. This observation points to the non-viable preparation of probiotics such as probiotics’ cell-free supernatants (CFS) as a valid and safer alternative to the use of live bacteria as anti-infective bio-therapeutics [14,15,16].

Others and we have demonstrated that most of the antimicrobial potential of CFS from lactic acid bacteria is due to the presence of organic acids, as their alkalization with NaOH results in a great reduction in their antibacterial effect, while heat treatment does not exert a significant effect [17,18,19]. Organic acids, such as lactic acids, act mainly by penetrating the cytoplasmic membrane and reducing the intracellular pH with the subsequent disruption of the transmembrane proton motive force, but the ability of lactic acid to permeabilize the outer membrane of Gram-negative bacteria has also been reported [20]. In addition to organic acids, proteinaceous components may also contribute to the antibacterial/antibiofilm activities of CFS, as digestion with proteases may reduce their effect, largely depending on the species/strain considered [18,19]. Such proteinaceous components include bacteriocins whose main mechanisms of action involve pore formation in bacterial membranes [14]. Interestingly, the ability to reduce biofilm formation or even disrupt biofilms has been reported for lactic acid bacteria and their supernatants due to several, and not yet fully elucidated, mechanisms, which include the inhibition of quorum sensing and virulence factor expression, inhibition of motility, extracellular DNase activity, reduction in the amount of the biofilm extracellular matrix, and modification of the bacterial cell surface, among others [14,21,22].

Among probiotics, *Lactobacillus plantarum* has been one of the most investigated for its effects against wound pathogens both in in vitro and in vivo experimental models [11,23,24,25,26]. Interestingly, the topical application of *L. plantarum* on human-infected chronic wounds has also been reported with promising results [25,26], although large controlled clinical trials involving a large number of patients are necessary to definitively establish the clinical effectiveness and safety of such an intervention.

Much less is known about *Lacticaseibacillus rhamnosus*, another widely used probiotic, in the context of wound infections. This latter probiotic was demonstrated to enhance keratinocyte viability in the presence of *S. aureus* when used in the form of either viable cells, cell-free lysates, or spent culture fluid [27] and to inhibit the growth and protease production of *P. aeruginosa* clinical strains isolated from wound samples [28]. Interestingly, the same probiotic was recently loaded in a bio-mimicking hydrogel and demonstrated an ability in vivo to significantly inhibit *P. aeruginosa* infection and inflammation and to promote superbacteria-infected wound healing [29].

Despite these promising results, scientific evidence for the therapeutic use of probiotics or their metabolites in wound care needs to be further supported by experimental data. In this regard, in the present study, we aimed at testing the antibiofilm activity of CFS from *L. rhamnosus* GG in a 3D wound infection model strictly resembling the conditions found in an infected wound [6,30]. Activity against both mono-species and dual-species biofilms of *P. aeruginosa* and *S. aureus* wound isolates was assessed, demonstrating the ability of CFS to rapidly and markedly kill biofilm-embedded bacteria as well as bacteria released from the biofilm. Importantly, differently from a conventional antibiotic, CFS failed to induce bacterial resistance following numerous passages at sub-inhibitory concentrations. Overall, these results strengthen the current view that probiotic-derived substances, also referred to as postbiotics, may represent promising alternatives to antibiotics, paving the way for their application as innovative tools in wound care.

## 2. Results

### 2.1. Killing Effect of Different Concentrations of L. rhamnosus GG CFS on Pre-Formed Biofilms at 24 h

**Figure 1:**
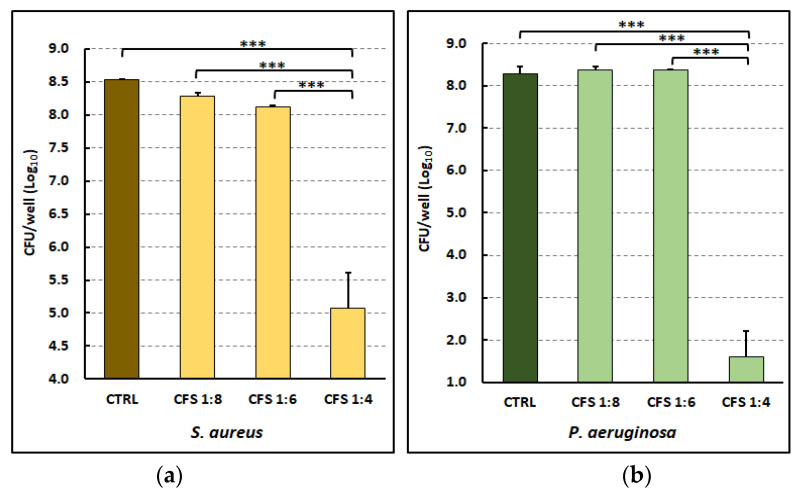
Effect of CFS from *L. rhamnosus* GG against mature biofilms following 24 h treatment. (**a**) Viable biofilm-associated count of the clinical isolate *S. aureus* W4; (**b**) viable biofilm-associated count of the clinical isolate *P. aeruginosa* W4. One-way ANOVA test followed by Tukey–Kramer multiple comparisons test. *** *p* < 0.001 vs. control (CTRL).

The killing effect of different concentrations of CFS was first tested against *P. aeruginosa* and *S. aureus* mono-species biofilms. To this aim, CFS were obtained by growing *L. rhamnosus* GG in De Man–Rogosa– Sharpe broth (MRSB) for 48 h at 37 °C. Bacterial cultures were then centrifuged, and the supernatants were passed through 0.22 µm filters. CFSs were diluted to 1:4, 1:8, and 1:16 in a simulant wound fluid (SWF) and used to treat pre-formed biofilms of both species developed on collagen scaffolds in the presence of SWF. The control wells contained MRSB diluted to 1:4 in SWF. Following 24 h of incubation at 37 °C, biofilm-embedded viable bacteria were evaluated via CFU count. As shown in Figure 1, a marked reduction in the cell viability of both strains was observed at 1:4 dilution. At such a concentration, far more than three orders of magnitude reduction in the CFU count was observed for both strains as compared to the untreated controls, demonstrating a net bactericidal effect in in vivo-like conditions.

The measurement of the pH of the biofilm culture medium mixed with different doses of CFS was performed at time 0 and following 24 h of biofilm treatment. As shown in Supplementary Figure S1, the pH values of CFS at time 0 decreased as the concentration of CFS increased from 1:8 to 1:4. The same figure also shows that the metabolic activity of the biofilm affected the pH of the cultures within 24 h, with *P. aeruginosa* promoting a marked alkalization of the biofilm medium and *S. aureus* an acidification in untreated samples. Only the treatment of biofilms with CFS 1:4 was able to ensure a pH value of the biofilm cultures close to 4 pH units, which correlated with the marked antibiofilm effect observed at such dilution. This observation suggests that ability of CFS to reduce pH below a certain level could be one of the factors contributing to their killing effect.

### 2.2. Killing Kinetics of CFS Diluted 1:4 in SWF against Biofilm-Embedded and Biofilm-Released Bacteria

**Figure 2:**
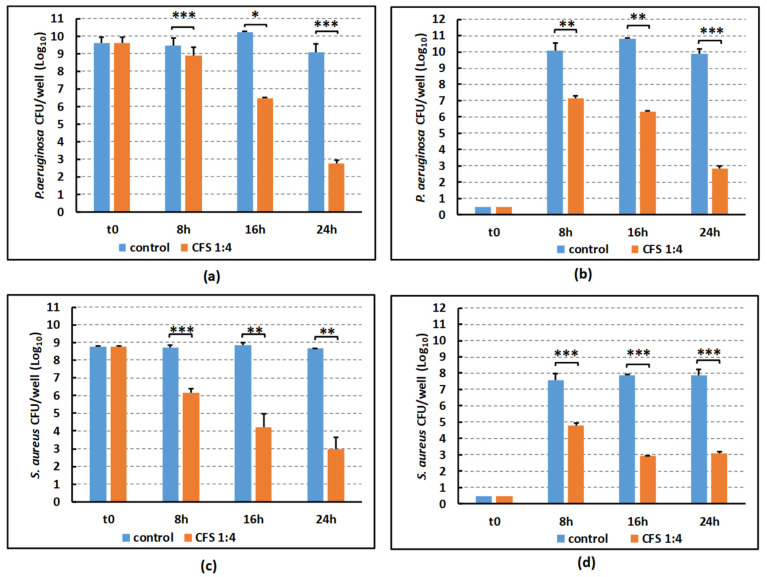
Activity of CFS against mono-species biofilms grown on collagen matrices in the presence of SWF. CFU numbers of *P. aeruginosa* W4 (**a**) or *S. aureus* W4 (**c**) embedded into the biofilm. CFU numbers of *P. aeruginosa* W4 (**b**) or *S. aureus* W4 (**d**) released from biofilm into the culture supernatants following treatment with CFS. *P. aeruginosa* or *S. aureus* biofilms formed on collagen scaffolds were treated for 8 h, 16 h, and 24 h with CFS diluted 1:4 in SWF (orange bars) or with MRSB diluted 1:4 in SWF as the control (blue bars). At each time point, the CFU count of control samples was compared with that of the treated ones by Student’s *t*-test. * *p* < 0.05, ** *p* < 0.01, *** *p* < 0.001. The graphics depict mean values ± SEM obtained in two experiments in duplicates for *P. aeruginosa* and in three experiments in duplicates for *S. aureus*.

We next evaluated the killing kinetics of CFS diluted 1:4 in SWF against both biofilm-embedded bacteria and bacteria detached from the biofilm and released in the biofilm supernatant. To this aim, preformed mono-species biofilms of *P. aeruginosa* and *S. aureus* were obtained in the 3D wound infection model and treated with *L. rhamnosus* GG CFS diluted 1:4 in SWF for 8 h, 16 h or 24 h. The viable counts in the supernatant (bacteria released from the biofilm) and in the biofilm (biofilm-embedded bacteria) were evaluated via CFU count. As shown in Figure 2a,c, in the control biofilms the number of biofilm-embedded bacteria remained quite stable over 24 h. In contrast, a time-dependent antibiofilm effect was observed against biofilm-embedded *S. aureus* and *P. aeruginosa* following treatment with 1:4 CFS. In the case of *S. aureus* (Figure 2c), an evident killing effect towards biofilm-embedded bacteria was obtained already at 8 h of incubation with approximately 2 logs reduction in the number of CFU as compared to the control at such time point. With regards to *P. aeruginosa* (Figure 2a), the killing kinetics was initially slower than against *S. aureus*, reaching 2 logs reduction between 8 and 16 h of incubation. Nevertheless, at 24 h a strong antibiofilm effect was observed with approximately 6 logs reduction in the CFU number of biofilm-embedded bacteria. High number of viable bacteria of both strains were released in the supernatant already at 8 h of incubation (Figure 2b,d). CFS diluted 1:4 were able to kill cells dispersed from the biofilms as well with a time-dependent kinetics.

### 2.3. Antibiofilm Effect of CFS on Dual-Species Biofilms

**Figure 3:**
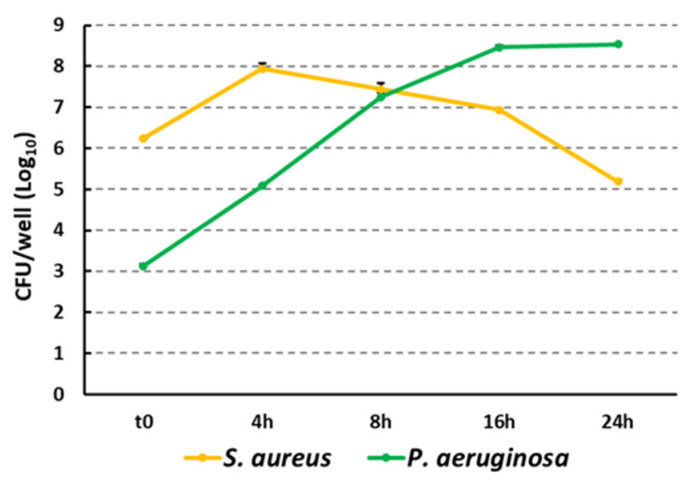
Growth kinetics of *S. aureus* and *P. aeruginosa* during dual-species biofilm formation in the 3D collagen wound infection model. Results from a representative experiment performed in duplicate are depicted.

*S. aureus* and *P. aeruginosa* often coexist in multi-species biofilms in chronic wounds, and their association can result in higher virulence and increased tolerance to antimicrobial agents [31]. Therefore, the killing effect of CFS diluted to 1:4 was also tested on dual-species biofilms formed on the collagen scaffolds. Several *S. aureus*–*P. aeruginosa* inoculation ratios were tested in preliminary experiments. The 1000:1 ratio was chosen as it allowed the predominant growth of *P. aeruginosa* towards *S. aureus* to be mitigated, yielding dual-species biofilms in which both species were adequately represented. As shown in Figure 3, although at time zero the *S. aureus* viable count was 1000 times higher than that of *P. aeruginosa*, the latter grew rapidly in the wound model, outnumbering *S. aureus* following 8 h of incubation. The 16 h co-incubation time was chosen to carry out the treatment with the CFS diluted 1:4 as at this time point, the dual-species biofilm was considered sufficiently mature, allowing the two species to be both represented and interacting with each other.

**Figure 4:**
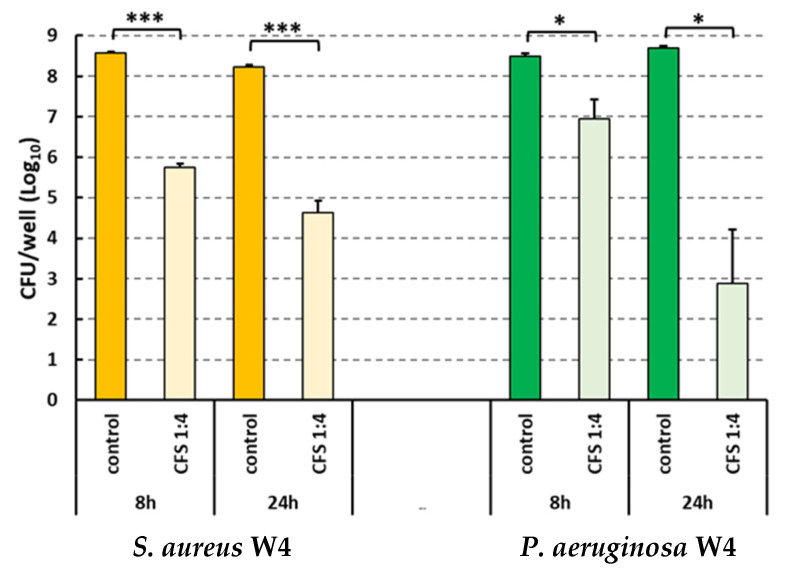
CFU numbers of bacteria recruited from dual-species biofilm following treatment with 1:4 CFS in SWF. Controls represent untreated biofilms (incubated with sterile MRSB diluted 1:4 in SWF). Mean values ± SEM of two experiments performed in duplicates are reported. * *p* < 0.05; *** *p* < 0.001, Student’s *t*-test, treated samples vs. controls.

At 16 h of co-incubation, dual-species biofilms were washed to remove unattached bacteria and treated with CFS diluted 1:4 in SWF. The effect of treatment was assessed following a further 8 and 24 h of incubation. At such time points, the biofilm-embedded viable counts of both *S. aureus* and *P. aeruginosa* were detected by performing CFU counts on selective agar plates. Results are reported in Figure 4. The numbers of both *S. aureus* and *P. aeruginosa* recruited from mixed biofilms were significantly reduced at both 8 h and 24 h of treatment as compared to untreated controls. As evident in Figure 4, in this experimental setup, *S. aureus* and *P. aeruginosa* coexisted in control samples in high numbers until the end of the incubation period. It is likely that the washings of the biofilms carried out at 16 h may have removed *P. aeruginosa*’s secreted components that are responsible for the inhibitory effect against *S. aureus*. On the other hand, *P. aeruginosa* biofilms seem to reach a steady state following 16 h of incubation that may have allowed *S. aureus* to growth and coexist with *P. aeruginosa*, unlike what was observed in Figure 3.

**Figure 5:**
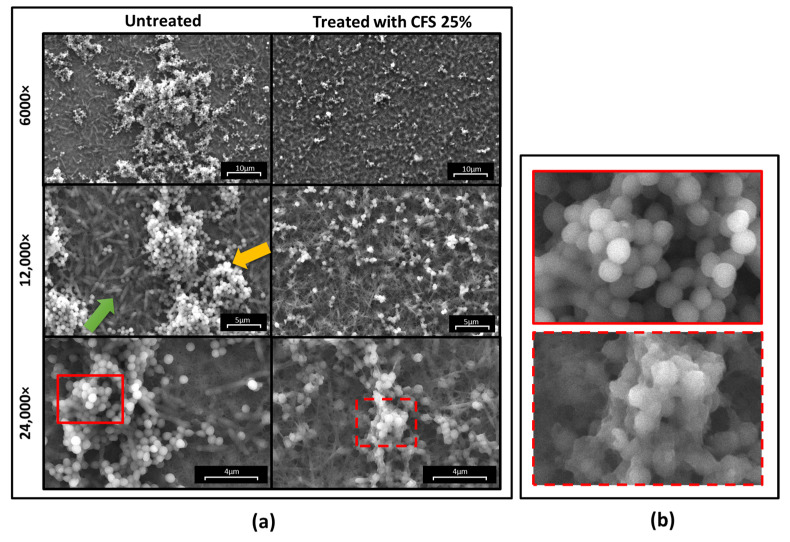
SEM images of *P. aeruginosa*–*S. aureus* dual-species biofilms grown in the collagen model in the presence of SWF. (**a**) Untreated and treated biofilms at different magnifications; (**b**) zoom of inserts highlighted in panel (**a**) to show the effect of treatment on bacterial morphology. Yellow arrow: *S. aureus* W4 microcolonies; green arrow: layer of *P. aeruginosa* W4.

The marked antibiofilm effect of CFS was confirmed by scanning electron microscopy of dual-species biofilms performed at 24 h treatment (Figure 5). While in untreated samples (samples incubated with SWF added with 1:4 sterile MRSB), numerous bacterial clusters of *S. aureus* were evident in the form of distinct micro-colonies (yellow arrow) laying over a continuous layer of *P. aeruginosa* cells (green arrow), in treated samples, a marked reduction in the number and size of micro-colonies of *S. aureus* was observed (Figure 5a). Furthermore, the continuous carpet of *P. aeruginosa* cells on the bottom had almost completely disappeared, making the collagen fibers visible. A marked alteration of *S. aureus* morphology was also evident, with the bacterial cells taking on a wrinkled appearance (Figure 5b).

### 2.4. Cytotoxic Potential of CFS against Human Keratinocytes

**Figure 6:**
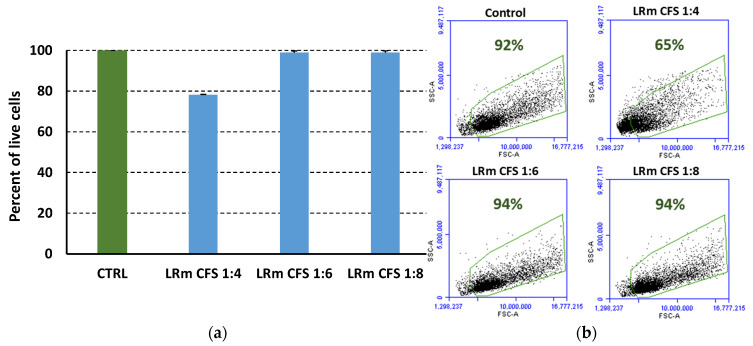
Flow cytometric determination of the cytotoxic effect of *L. rhamnosus* GG cell-free supernatants (LRm CFS) on human keratinocytes. (**a**) Live cell percentages following 24 h exposure to LRm CFS diluted 4, 6, or 8 times with complete DMEM; (**b**) morphological flow cytometric analyses where numbers indicate cell percentages in the healthy cell gate (green delimited) (CTRL, Control: cells grown in DMEM, 25% MRSB). Mean values ± SEM are shown, *n* = 3.

To further explore the translational potential of *L. rhamnosus* GG CFS, the cytotoxic activity of the CFS diluted 4, 6, and 8 times in complete DMEM medium was tested in vitro against the human keratinocyte cell line HaCaT grown on the surface of collagen scaffolds in the 3D wound model. Following a 24 h incubation, cytotoxic activities of less than two percent were observed for 1:6 and 1:8 diluted CFS, while when CFS was diluted 1:4, the cytotoxicity was around 20% (Figure 6a). Moreover, when cell morphology analyses of the HaCaT cells were performed on forward vs. side scatter plots by using a flowcytometer, only a slight to mild degree of morphological changes was observed at all the dilutions tested (Figure 6b).

### 2.5. Induction of Resistance by CFS as Compared to Ciprofloxacin

**Figure 7:**
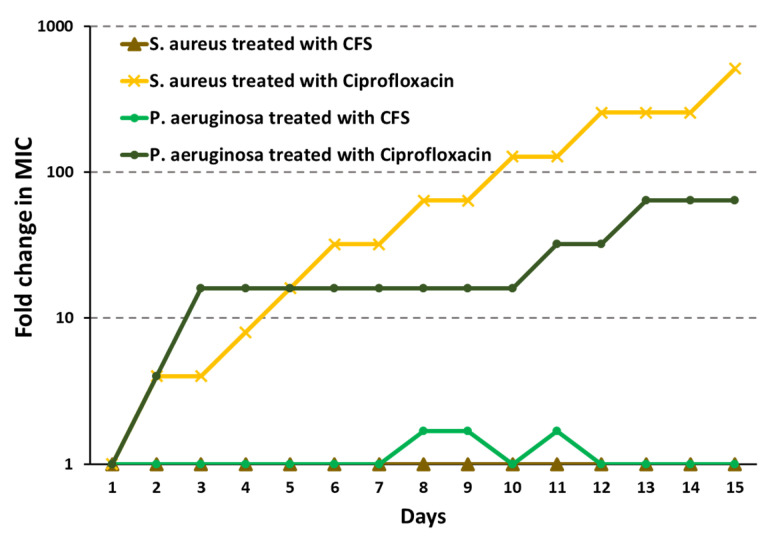
Induction of resistance by CFS as compared to ciprofloxacin against *P. aeruginosa* W4 and *S. aureus* W3 clinical isolates evaluated as MIC changes. Bacteria were exposed to 0.5 × MIC concentration of CFS or ciprofloxacin daily for 15 consecutive passages.

The tendency of inducing antimicrobial resistance by ciprofloxacin and CFS against both *P. aeruginosa* W4 and *S. aureus* W3 was monitored by determining the MIC values of the antibiotic and CFS respectively, during 15 days of consecutive treatment of the bacteria at a concentration of 0.5 × MIC (Figure 7). For the purpose of these experiments, the clinical isolate *S. aureus* W3 was used instead of S. aureus W4, as the latter was resistant to ciprofloxacin. The MIC of ciprofloxacin rapidly increased to above 10-fold following as little as 3 and 5 passages for *P. aeruginosa* and *S. aureus*, respectively. In 15 days, the MIC of ciprofloxacin had increased 512-fold against *S. aureus*, while that of the same antibiotic against *P. aeruginosa* had increased 64-fold. In contrast, only slight and temporary variations in the MIC values of CFS were observed especially against *P. aeruginosa* over 15 days, not rising above 2-fold. This finding suggested that the tendency of CFS to induce resistance was very low.

## 3. Discussion

Infection is one of the most frequent complications of non-healing wounds, but the bacterial tendency to grow as a biofilm, inefficient host defense, and resistance to antibiotics result in a considerable decrease in the possibility of effectively treating infected wounds [5]. Although mechanical debridement is regarded as a safe, quick, and relatively inexpensive technique to remove biofilms from chronic wounds, it is often insufficient to remove all the biofilm-associated bacteria, and a combined approach involving the use of antibiofilm agents is needed [5]. Antiseptics such as silver compounds are commonly used as topical agents for chronic wounds, but they are relatively toxic to keratinocytes in in vitro studies [32]. On the other hand, topical antibiotics can induce contact allergy and delayed-hypersensitivity reactions and could more likely lead to the development of resistance than use of systemic antibiotics. Given the drawbacks of current therapies, alternative approaches are widely demanded.

In the present study, we aimed to test the antibiofilm activity of a cell-free extract of *L. rhamnosus* GG in conditions resembling those found in an infected wound. Although probiotics or their products are attracting considerable interest as innovative bio-therapeutics in wound care, most of the available in vitro studies test their effects against wound pathogens in basic laboratory conditions, by using standard agar well-diffusion assays or the microtiter plate assay for studying biofilm formation [24,33,34]. A panel of global wound biofilm experts has recently highlighted the importance of employing in vivo-like systems to more adequately predict the efficacy in vivo of new antibiofilm treatments [5]. The same panel identified the following points, which are clinically relevant in vitro methodologies: (i) use media containing serum or blood protein; (ii) use mature biofilms; (iii) test single and polymicrobial cultures; and (iv) show measurable reduction in biofilm bacterial count over a clinically relevant time [5].

All these points have been met in our antibiofilm assay. Biofilms were grown in a SWF containing serum proteins that also are present in the wound environment. Furthermore, biofilms were developed on the surface of a collagen type I simulating the semi-solid matrix of the wound bed and not on a well-defined solid surface, as is the case when using a standard polystyrene well plate as the substrate for biofilm formation. This is an important aspect as it has been demonstrated that both the nutritional composition of the culture medium and the type of substrate can influence biofilm development by bacterial pathogens [35,36]. The conditions adopted in the present study have been previously reported to allow the formation of bacterial aggregates (e.g., biofilms) very similar to those found in tissue biopsies from chronically infected wounds [30], suggesting that such conditions were indeed suitable to simulate the wound environment. In such in vivo-like conditions, our study demonstrated that CFS from *L. rhamnosus* GG displays a marked ability to reduce the viable bacterial load associated with mature mono-species biofilms of both *P. aeruginosa* and *S. aureus* already at 8 h treatment. We have recently reported that the acidity of *L. rhamnosus* GG CFS, whose pH is 3.87 ± 0.055, plays a major role in its antibacterial effect as this is almost completely abrogated when CFSs are adjusted to pH 6.0 with NaOH, while it is maintained after heating (at 70 and 100 °C for 30 min) [17]. In addition, preliminary data obtained by us fractionating CFS in several fractions on the basis of the molecular weight revealed that most, though not all, of the bactericidal effect of CFS is retained in the low molecular weight fraction (<3 KDa) (our unpublished observation). As this fraction is also the one likely containing small organic acids, overall, these observations suggest that organic acids rather than heat-sensitive components are mainly responsible for the CFS antibacterial effect. In this study, the antibiofilm effect of the same *L. rhamnosus* GG CFS was almost exclusively evident at a dilution in SWF of 1:4 (Figure 1), which allowed the maintenance of a net acidic pH in the biofilm environment for the treatment duration, thus ensuring CFS optimal activity. Interestingly, the killing kinetics against *S. aureus* appeared to be slightly more rapid than against *P. aeruginosa*. This might be due to the previously reported ability of *P. aeruginosa* to produce ammonia during growth [37] that, at early exposure times (i.e., before the bacterial load starts to decrease), could partially neutralize the acidic environment, thus impacting CFS activity. Accordingly, our study demonstrated that, in the adopted wound-like conditions, untreated *P. aeruginosa* biofilms caused a net increase in the pH (see Supplementary Figure S1, control samples), an observation in strong agreement with the widely reported alkaline environment of chronic wounds (within the range of 7.15–8.9), of which *P. aeruginosa* is a major pathogen [38,39]. Actually, pH has a great impact on the wound environment, influencing many processes such as wound healing, oxygen release, angiogenesis, protease activity, and bacterial toxicity [39]. Specifically, alkaline pHs have been reported to negatively impact wound healing, favor the activity of the host as well as bacterial proteases, and inhibit oxygen release [38,39]. The pH of the wound shifts towards neutral and becomes acidic after complete healing [40]. The modulation of wound pH toward acidic values by CFS could therefore exert a therapeutic effect not only by reducing the bacterial bio-burden but also by promoting healing and tissue oxygenation, as optimal oxygen release from hemoglobin occurs at low pH. Evidence suggests that the recognized efficacy of honey (whose pH typically ranges from 3 to 4) as a wound care agent is greatly related to its acidification ability [41]. On the other hand, studies in animal models have demonstrated that the topical acidification of wounds results in beneficial effects promoting epithelialization, collagen deposition, and healing, with best outcomes observed with solutions at pH 3.5–4.0 [42,43]. For all these reasons, the results obtained herein support the therapeutic use of acidic *L. rhamnosus* GG CFS in wound care, providing experimental evidence for the encouraging results obtained in few clinical trials that used topical lactobacilli or their supernatants for the management of infected wounds [25,44].

Biofilm formation is a complex and multistep process that involves three main phases: adhesion, maturation, and dispersal [45]. This latter consists in the constant release of single cells or clusters of cells that return to the planktonic state and colonize surrounding area to establish novel biofilms, thus representing a crucial step in the diffusion of the pathogenic process at the wound site [46]. It has been previously demonstrated that bacterial cells dispersed from a biofilm represent a distinct stage in the transition from bacterial biofilm to planktonic lifestyle [47]. Specifically, they maintain some features specific to the biofilm mode of growth, such as a more virulent and resistant phenotype, and exhibit gene expression patterns that are distinct from both exponential and stationary-phase planktonic cells [48]. In this study, we therefore investigated the ability of CFS to target not only biofilm-embedded bacteria but also biofilm-derived planktonic cells growing in a wound-like environment. We demonstrated that CFS efficiently killed dispersed cells, reducing the viable count of this unique and important phenotype by several orders of magnitude, further supporting the potential role of CFS for clinical applications at the wound site. The result was not obvious, as it has been previously reported that, depending on the dispersion cue, dispersed cells from *P. aeruginosa* biofilms acquire a transient resistance to colistin and require approximately 2 h to revert to a susceptible phenotype [48].

It is widely agreed that wound infections are often polymicrobial [4]. Thus, we proceeded to test the antibiofilm activity of *L. rhamnosus* GG CFS against *S. aureus* and *P. aeruginosa* dual-species biofilms. These two species establish complex interactions that result in augmented virulence and the formation of strong dual-species biofilms that maintain the chronic infection, impairing the healing of the wound and increasing the development of antibiotic resistance [4]. When co-cultured in our wound model, both species underwent evident and rapid growth. Nevertheless, despite the fact that the *S. aureus*–*P. aeruginosa* ratio at time 0 was 1000:1, *P. aeruginosa* outnumbered *S. aureus* after 8–16 h of co-culture. These results are in agreement with previous studies that demonstrated *P. aeruginosa* killing ability towards *S. aureus* when co-cultured in planktonic form in standard laboratory media or in sputum obtained from patients with cystic fibrosis [49,50]. The killing ability of *P. aeruginosa* on *S. aureus* has been attributed to different extracellular factors secreted by the former during growth, including anti-staphylococcal 4-Hydroxy-2-Heptylquinoline N-Oxide (HQNO), pyocyanin, and LasA protease [51]. Despite the likely inter-species competitive interaction, at 16 h of co-culture in our biofilm model, the two species coexisted in a mature dual-species biofilm and were subjected to CFS treatment, which significantly reduced the bioburden of both species at both 8 and 24 h, as assessed by CFU counting and electron microscopy imaging. Interestingly, such imaging revealed a non-random distribution of the two species in the control samples, with *P. aeruginosa* residing mainly in a continuous deeper layer over the collagen matrix or infiltrating within the collagen fibers and *S. aureus* forming discrete micro-colonies located in the most superficial layers of the biofilm. Such a distribution resembles that found in human wound biopsy samples or that described in other in vitro wound models, and might reflect *P. aeruginosa*’s ability to move toward the deeper layers of the wound, where access to nutrients and space availability might be less competitive [52,53,54]. In the treated samples, SEM images revealed a marked reduction in the number and size of micro-colonies and an evident damage of bacterial morphology, confirming the data of the CFU count.

CFS toxicity evaluation against the immortalized human keratinocyte cell line HaCaT revealed that, at the active antibiofilm concentration of 1:4, CFS caused the membrane permeabilization of approximately 20% of the cells, as assessed by PI uptake and flow cytometry [55]. Such a level of cytotoxicity is within the cut-off point indicated by the ISO 10993-5:2009(E) [56] protocol, which considers cytotoxic to be a reduction in cell viability by more than 30%. Furthermore, a qualitative morphological evaluation of CFS cytotoxicity performed by flow cytometry analysis of forward versus side scatter (size vs. granularity) revealed no major difference with untreated cells, suggesting a mild grading of reactivity according to the ISO 10993-5:2009(E) protocol.

The cytotoxic potential of lactobacilli CFS towards host cells is still a relatively poorly investigated subject. Supernatants of *L. gasseri* and of *L. crispatus* were found to be cytotoxic against the HeLa tumor cell line independently from pH and lactate but not toward the human normal fibroblast-like cell-line HNCF-PI 52 [57]. Less than 10% cytotoxicity was observed when exposing human hepatocellular carcinoma HepG2 cells to CFS from several *Lactobacillus* strains [58]. Further research is needed to elucidate the specific mechanisms and conditions under which lactobacilli supernatants may exert cytotoxic effects and to find possible solutions to minimize such effects on host cells. For instance, we have previously reported that the encapsulation of a relatively toxic antimicrobial peptide in chitosan-based nanoparticles was able to greatly reduce peptide’s cytotoxic potential towards mouse embryo fibroblast cells without altering its antibacterial properties that were, instead, reinforced by the chitosan carrier itself [59]. Sharaf and co-workers recently employed a similar strategy, loading CFS in nano-chitosan and obtaining promising results [60]. Thus, the development of appropriate CFS carriers/formulations is worth intensive research for likely increasing their applicative potential. It is also worth mentioning that silver-based wound dressings are widely used in wound management, although high levels of toxicity have been demonstrated against keratinocytes and fibroblasts in monolayer cultures for silver nitrate solution and nanocrystalline silver released from a commercially available dressing [32]. No cytotoxic effect of *L. rhamnosus* GG CFS was recently reported by us in an in vivo *Galleria mellonella* model [17]; likewise, no evident adverse effect was reported in few patients with second-degree burn wounds topically treated with lactobacilli CFS [44]. These observations suggest that at the tissue level, eventual CFS cytotoxicity might be mitigated, a hypothesis supported also by the reported ability of probiotics to promote wound re-epithelization, neovascularization, and wound healing with no adverse effects [61].

One interesting aspect investigated in our study was the induction of resistance by CFS following multiple bacterial exposure to sub-inhibitory concentrations, an aspect that to the best of our knowledge has never been reported before. Ciprofloxacin was chosen as a comparison, as it is an antibiotic used in the treatment of wound infections doubly infected with *P. aeruginosa* and *S. aureus* [4]. While ciprofloxacin rapidly induced resistance in both *P. aeruginosa* and *S. aureus*, susceptibility to CFS remained unchanged following 15 passages at sub-inhibitory concentrations, strengthening the applicative potential of CFS as a valid alternative to antibiotics in the management of infected wounds. We have previously provided evidence that the antibacterial action of CFS is most likely linked to the presence of organic acids [17]. Thus, the lack of resistance induction by CFS could be due to the reported ability of organic acids to destabilize bacterial membranes [20]. Indeed, this mechanism of action is less prone to induce resistance than antimicrobials having action targets more easily undergoing spontaneous mutations or acquired changes, as is the case with ciprofloxacin [62].

## 4. Materials and Methods

### 4.1. Bacterial Strains and Growth Conditions

The bacterial strains used in the study were isolated from infected wounds at the Microbiology Unit of Pisa University Hospital, Italy, following standard microbiological procedures and are now part of a collection of strains in the Microbiology section of the Department of Translational Research and New Technologies in Medicine and Surgery at the University of Pisa. The Phoenix System (Becton Dickinson Italia; Milan, Italy) was used for antibiotic susceptibility testing. The isolates from both Gram-positive and Gram-negative species utilized in this study displayed different drug susceptibility profiles (Table 1). For the preparation of stock cultures, bacterial strains were grown in Tryptic Soy Broth (TSB, Oxoid, UK) until the late-log phase, subdivided into aliquots, and kept frozen at −80 °C until required.

### 4.2. Preparation of CFS of Lacticaseibacillus rhamnosus GG

*Lacticaseibacillus rhamnosus* GG was isolated from a commercial dietary supplement (Microbiosys, Sanofi, Vitry-Sur-Seine, France). Identification of the isolate at the species level was assessed by MALDI-TOF-MS as previously described [17]. Novogene (Beijing, China) performed strain identification through whole genome sequencing and data analysis. A mapping rate of 99.83% with strain GG’s reference genome was obtained. An overnight culture of *L. rhamnosus* GG grown in MRSB (Oxoid, Basingstoke, Hampshire, UK) was diluted 1:100 in fresh MRSB and incubated at 37 °C for 48 h. Following incubation, the culture was centrifuged at 4000× *g* for 10 min and culture supernatants were collected and filtered through 0.22 µm filters. No alkalization of CFS was carried out before the assays, as we have previously demonstrated that such treatment abrogates almost completely the CFS antibacterial effect against *P. aeruginosa* [17]. CFS aliquots were kept at −20 °C and defrosted before use.

### 4.3. 3D In Vitro Wound Infection Model

For the 3D in vitro wound infection model, collagen scaffolds containing polymerized collagen in a SWF were formed to mimic an in vivo wound bed as previously described with minor modifications [6]. Briefly, the rat tail collagen type 1 (Corning, MA, USA) was diluted 1:5 on ice with SWF consisting of 50% fetal bovine serum (Euroclone S.p.a, Milan, Italy) and 50% peptone water (0.1% peptone in 0.85% sodium chloride (NaCl), Merck, Milan, Italy). The pH of the solution was adjusted to 8.5 with NaOH (1N) to mimic the conditions found in a chronic wound [38]. A quantity of 200 µL of the collagen solution was layered on each well of a 24-well plate and incubated at room temperature for 1 h to allow polymerization of the 3D collagen matrix. Polymerized collagen scaffolds were subsequently infected with bacteria to form biofilms.

### 4.4. Mono-Species and Dual-Species Biofilm Formation on Collagen Scaffolds

For mono-species biofilm formation, overnight cultures of bacterial suspensions of *P. aeruginosa* W4 or *S. aureus* W4 were diluted in SWF to reach a bacterial load of approximately 2 × 10^5^ CFU/mL. A quantity of 75 µL of each bacterial suspension was inoculated into wells containing collagen matrices. Following 24 h of incubation at 37 °C, wells were washed 3 times with NaCl 0.9% to remove non-adherent bacteria and subjected to CFS treatment (paragraph 4.5).

An optimized *P. aeruginosa* W4–*S. aureus* W4 ratio of 1:1000 was chosen as inoculum for dual-species biofilm formation based on preliminary experiments. To this aim, *P. aeruginosa* W4 and *S. aureus* W4 overnight cultures were diluted in SWF to reach bacterial densities of 4 × 10^7^ and 4 × 10^4^ CFU/mL, respectively. A quantity of 40 µL from each bacterial suspension were mixed, layered onto the surface of collagen scaffolds, and incubated at 37 °C. Biofilms were left untouched for different intervals of times, i.e., without changing the medium above the biofilms or removing bacteria released from the biofilm into the biofilm supernatants. At time 0, 4 h, 8 h, 16 h and 24 h post-infection, biofilms were washed 3 times with NaCl 0.9% to remove non-adherent bacteria and CFU were enumerated as explained in Section 4.5.

### 4.5. Mature Biofilms Treatment with CFS and Bacterial Enumeration

The antibiofilm activity of CFS against mature (24 h old) mono-species biofilms was first evaluated following a 24 h treatment. To this aim, CFS were diluted 1:4, 1:6, and 1:8 times in SWF and 500 µL of the diluted solutions were added to the biofilm-containing wells. No pH adjustment was made to the diluted samples, in order to fully account for the effect of dilution on the antibacterial effect. Controls consisted of biofilms incubated with 500 µL SWF containing MRSB 1:4. Following 24 h incubation at 37 °C, treated and control biofilms were washed 3 times with NaCl 0.9% to remove non-adherent bacteria, and processed for bacterial enumeration. To this aim, 300 µL of PBS were added to the washed wells and the collagen scaffolds were disrupted with the cut end of a sterile 1 mL pipette tip. The pieces of collagen were collected in microtubes. The procedure was repeated once to collect all the collagen pieces in the wells (total volume of PBS 600 µL). Collected collagen pieces in microtubes were dissolved by adding 600 µL of collagenase type I (MP Biomedicals, Solon, OH, USA) dissolved in PBS solution at a concentration of 500 µg/mL. After 1 h of incubation at 37 °C in shaking conditions (600 rpm) and vigorous vortexing for 1 min, collagenase was washed away by centrifuging at 4000× *g* for 10 min and re-suspending the pellet in PBS. Bacterial suspensions were serially diluted and plated on TSA for CFU count.

Killing kinetics of CFS against mono-species biofilms were then evaluated in the CFS dilution in SWF of 1:4, following 8 h, 16 h, and 24 h of treatment. In this case, both biofilm-embedded bacteria and bacteria released from the biofilms in the culture supernatants were enumerated. Specifically, 500 µL of the SWF–CFS treatment mixture containing planktonic bacteria released from the biofilms were collected at 8 h, 16 h, and 24 h of incubation. Biofilms were further washed with 300 µL of PBS, which were added to the corresponding 500 µL of the SWF–CFS mixture. Bacterial suspensions were vortexed for 1 min, serially diluted in PBS, and plated on TSA for CFU count. Biofilm-embedded bacteria were evaluated following 8 h, 16 h, and 24 h of treatment as explained above.

Dual-species biofilms (16 h old) were subjected to 3 washings to remove non-adherent bacteria and mixed with 500 µL of CSF diluted 1:4 in SWF. The effect of treatment was assessed following further 8 and 24 h of incubation (for a total of 40 h of incubation of the biofilms). Following treatment, biofilm-embedded bacteria were obtained as explained before, serially diluted, and plated on agar cetrimide (Merck, Milan, Italy) and TSA–7.5% NaCl for enumeration of *P. aeruginosa* and *S. aureus*, respectively.

### 4.6. Scanning Electron Microscopy Analysis of Dual-Species Biofilms

Imaging of dual-species biofilms grown on collagen matrices and treated for 24 h with 25% CFS in SWF or left untreated (incubated with 25% MRSB in SWF) was performed by scanning electron microscopy (SEM). The biofilm-containing collagen scaffolds were washed 3 times with sterile water and fixed with 2.5% glutaraldehyde at 4 °C for 2.5 h. The specimens were then subjected to increasing concentrations of ethanol (25%, 50%, 75%, 95%, and 100%) for dehydration. The dehydrated specimens were dried under a flow laminar hood overnight and examined by field emission SEM FEI Quanta 450 FEG from the Center for Instrument Sharing of the University of Pisa, Italy. Images were acquired from randomly selected areas on each sample using 6000×, 12,000×, and 24,000× magnifications.

### 4.7. Cytotoxicity Assay

Cytotoxic potential of the CFS from *L. rhamnosus* GG was tested against the human keratinocyte cell line HaCaT, kindly provided by Professor Serena Danti from the University of Pisa, Italy, in the in vitro 3D wound model. To this aim, cells were seeded on collagen scaffolds, formed on 0.33 cm^2^ well inserts with 0.4 μm diameter pores (TC-inserts, Sarstedt, Numbrech, Germany), at a density of 50,000 cells/well in high glucose Dulbecco’s Modified Eagle Medium (DMEM) mixed with 2 mM L glutamine and 10% fetal bovine serum (FBS) [complete DMEM]. The same medium without cells was added to the inferior chamber of the wells, as well. Cells on the upper chamber were incubated for 24 h at 37 °C in a humidified atmosphere containing 5% CO_2_ to reach approximately 90–100% confluence. Following washing to remove non-adherent cells, adherent cells were added with 200 μL of CFS diluted to 1:4, 1:6, and 1:8 in complete DMEM or with 200 μL of MRSB diluted 1:4 in complete DMEM (negative control) and incubated for 24 h at 37 °C in a humidified atmosphere containing 5% CO_2_. Following incubation, the wells were washed once with 200 μL warm PBS and the scaffolds digested with 5 mg/mL collagenase for 1 h at 37 °C. Following a centrifugation at 700× *g* to collect the cells, the pellets were subjected to trypsin/ethylenediaminetetraacetic acid (EDTA) treatment at 37 °C for 3 min to dissociate eventual cell aggregates. Following a wash with PBS (700× *g*) the pellets were resuspended in PBS. Cells were stained for 5 min with 0.1 μg/mL propidium iodide (PI, Merck, Milan, Italy) at room temperature before performing flow cytometric acquisition. To this aim, 50,000 events were acquired ungated by using a flow cytometer (BD Accuri C6, BD Biosciences, Italy). The percentage of PI-positive HaCaT cells was calculated by computer-assisted analyses (BD Accuri C6 software version 1.0.264.21, BD Biosciences, Milan, Italy) and the percent of vitality was calculated according to the following formula: 100 − [100 × (%PI pos sample − % neg control)]/(100 − % neg control). In addition, a morphological evaluation of treated HaCaT cells was performed by manually setting a healthy cells gate in the forward scatter versus side scatter plot, based on the morphology. The percentage of cells within the gate following treatments were calculated by the following formula: 100 × (number of cells within the gate following treatments/number of total cells).

### 4.8. MIC Determination and In Vitro Assessment of Resistance Induction by CFS

The MIC values of ciprofloxacin and CFS against *P. aeruginosa* W4 and *S. aureus* W3 were determined using a standard broth microdilution method. Briefly, ciprofloxacin (Bayer, Leverkusen, Germany) or CFS were serially diluted in Mueller Hinton Broth (MHB, Sigma-Aldrich) and MRSB, respectively, in 96-well plates (100 µL). Exponential phase cultures of *P. aeruginosa* W4 or *S. aureus* W3 were diluted in MHB to obtain 1 × 10^6^ CFU/mL, and 100 µL were added to each well. Plates were incubated at 37 °C for 20 h. The MIC was determined as the lowest concentration of antibiotic or CFS at which no visible growth was observed. Experiments were performed in triplicate.

For resistance induction assessment, bacteria grown at ½ MIC value were collected, diluted in fresh MHB to obtain 1 × 10^6^ CFU/mL, and added to ciprofloxacin or CFS serial dilutions, freshly prepared as described above. This procedure was performed every day for 15 consecutive days. At each passage MIC value were recorded and expressed as fold changes as compared to time 0.

### 4.9. Statistical Analysis

Each experiment was repeated at least three times in duplicates (*n* = 6) unless otherwise specified. GraphPad In Stat version 3.06 (GraphPad Software Inc., La Jolla, CA, USA) was used to assess the statistical significance of the data. One-way ANOVA followed by Tukey–Kramer’s multiple comparisons test was applied when assessing differences among three or more groups of data. Student’s *t*-test was employed when comparing two groups of variables. A *p* value < 0.05 was considered statistically significant.

## 5. Conclusions

Metabolites derived from probiotics seem to be a promising therapeutic/preventive strategy in the era of antibiotic resistance. Herein, we have demonstrated a strong anti-biofilm activity of CFS from *L. rhamnosus* GG in conditions closely resembling the wound bed and, to the best of our knowledge, for the first time, a lack of CFS resistance induction. Due to the acidic nature of the CFS, their topical use in antimicrobial wound therapy could not only target bacterial cells within or released from the biofilm but also correct the wound micro-environment, where the pH shift from acidic to alkaline in chronic wounds is consistently reported as an obstacle to wound healing.

## Figures and Tables

**Table 1:** Bacterial strains.

Bacterial Species	Strain Name	Isolation Site	Resistance Profile *
*Pseudomonas aeruginosa*	W4	Wound	Susceptible
*Staphylococcus aureus*	W4	Wound	AMP-CLI-ERY-FOF-GEN-OXA-PEN-TET-CIP
*Staphylococcus aureus*	W3	Wound	GEN

* CIP—ciprofloxacin; AMP—ampicillin; CLI—clindamycin; ERY—erythromycin; FOF—fosfomycin; GEN—gentamicin; OXA—oxacillin; PEN—penicillin; TET—tetracycline.

## Data Availability

The data presented in this study are available on request from the corresponding author.

## Supplementary Materials

The following supporting information can be downloaded at: https://www.mdpi.com/article/10.3390/ijms25042087/s1.
