# Supplementary material for: Strong Activity and No Resistance Induction Exerted by Cell-Free Supernatants from Lacticaseibacillus rhamnosus against Mono-Species and Dual-Species Biofilms of Wound Pathogens in In Vivo-like Conditions
Source: Int J Mol Sci. 2024 Feb 8;25(4):2087. doi: 10.3390/ijms25042087 (PMC10888627; doi:10.3390/ijms25042087)
Supplement: Supplementary file 1 [file ijms-25-02087-s001.zip › ijms-2824590-supplementary.pdf]

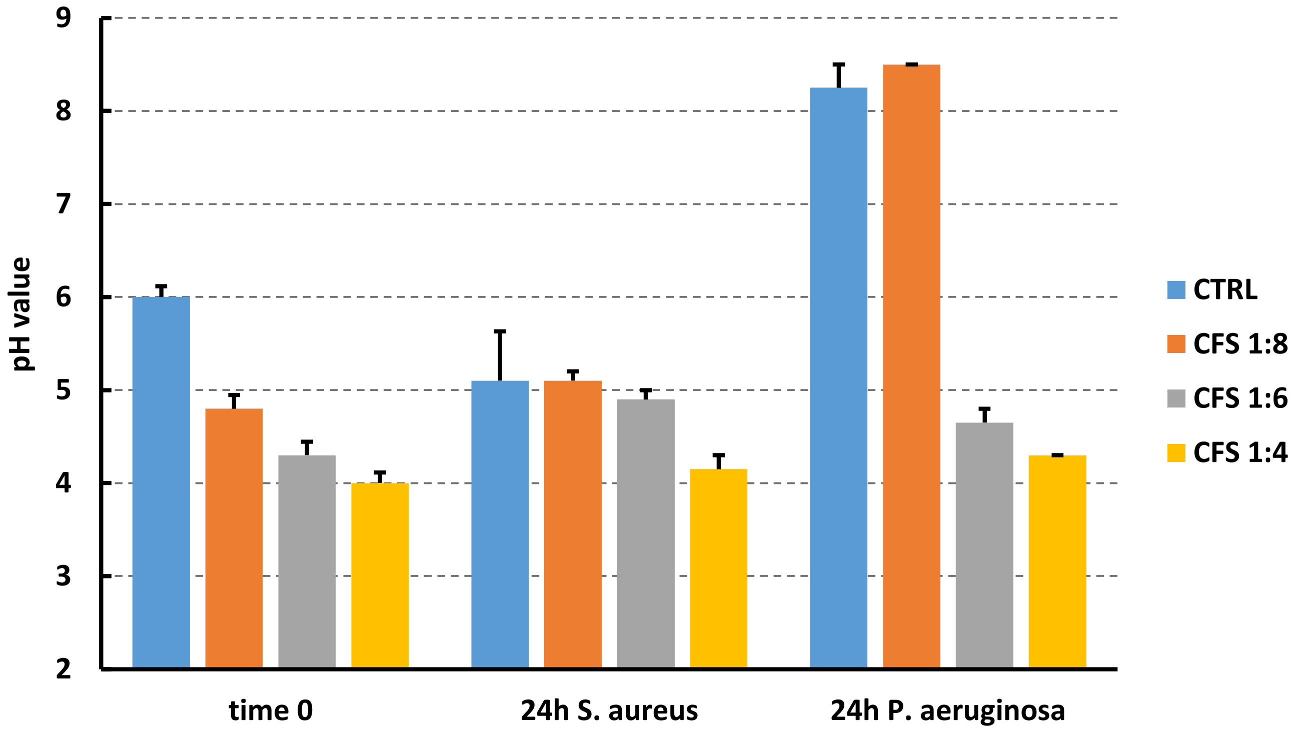

Supplementary Figure 1. pH values of different concentrations of CFS diluted in SWF at time 0 and following 24 h culture with pre-formed *S. aureus* W4 or *P. aeruginosa* W4 biofilms. CTRL: control samples incubated with SWF containing sterile MRSB diluted 1:4.
